# Ultrasonic Manipulation of Yeast Cells in Suspension for Absorption Spectroscopy with an Immersible Mid-Infrared Fiberoptic Probe

**DOI:** 10.1016/j.ultrasmedbio.2013.01.003

**Published:** 2013-06

**Authors:** Cosima Koch, Markus Brandstetter, Bernhard Lendl, Stefan Radel

**Affiliations:** Institute of Chemical Technologies and Analytics, Vienna University of Technology, Vienna, Austria

**Keywords:** Ultrasonic particle manipulation, Fourier transform infrared spectroscopy, Acoustic radiation force, Ultrasonic standing wave, Attenuated total reflection, *Saccharomyces cerevisiae*

## Abstract

Recent advances in combining ultrasonic particle manipulation with attenuated total reflection infrared spectroscopy of yeast suspensions are presented. Infrared spectroscopy provides highly specific molecular information about the sample. It has not been applicable to in-line monitoring of cells during fermentation, however, because positioning cells in the micron-thin measurement region of the attenuated total reflection probe was not possible. Ultrasonic radiation forces exerted on suspended particles by an ultrasonic standing wave can result in the buildup of agglomerates in the nodal planes, hence enabling the manipulation of suspended cells on the microscopic scale. When a chamber setup and a prototype in-line applicable probe were used, successful control over the position of the yeast cells relative to the attenuated total reflection sensor surface could be proven. Both rate of increase and maximum mid-infrared absorption of yeast-specific bands during application of a pushing frequency (chamber setup: 1.863 MHz, in-line probe: 1.990 MHz) were found to correlate with yeast cell concentration.

## Introduction

When an ultrasonic standing wave (USW) is applied to a suspension, radiation forces are exerted on the suspended particles. The origin of these forces are the spatial gradients of the sound wave's acoustic pressure (King 1934). Depending on the mass density and speed of sound of the particles and the host liquid, respectively, the particles are driven into regions of vanishing displacement or pressure. Therefore, the nodes within a standing wave are regions where particle aggregation (or thinning) can be observed; solid particles typically travel into the pressure nodes of the sound field. Acoustic radiation forces have previously been used for reliable sample concentration in sensor applications (Coakley 1997; Hill et al. 2004), including medical environments (Barnes et al. 1998). Furthermore, Saito et al. (2002) showed that locomotive particles could be manipulated/positioned by a combination of perpendicularly aligned ultrasonic standing wave fields. The ability of an USW to deposit particles on a surface has been investigated with functionalized surfaces (Hawkes et al. 2004) and by optical means (Glynne-Jones et al. 2009).

Mid-infrared (mid-IR) Fourier transform infrared (FT-IR) spectroscopy is a well-developed method in chemical analysis. In combination with attenuated total reflection (ATR) sensing elements, (mid-IR) spectroscopy appears to be a very promising method for process monitoring, particularly useful in (bio)process applications. The ATR technique exploits the occurrence of total reflection of light at the interface of two media with different refractive indices and, thus, only has a detection range of some microns, that is, within the evanescent field. New devices and concepts for advanced chemical analysis based on this technique have been developed over the years (Harrick 1967). For measurements in suspension, additional measures are necessary to bring a sufficient amount of suspended particles into the evanescent field region. Recently, the mid-IR spectroscopic assessment of biological cell sediment onto a horizontal ATR element was reported (Jarute et al. 2004; Schnöller and Lendl 2004). Sedimentation, however, is not an option for in-line measurements, for example, in bioreactors.

The recording of an FT-IR absorbance spectrum of a given sample consists of two successive measurements. In each measurement the intensity recorded at the detector for different wavenumbers $\tilde{\nu }$ (reciprocal λ, expressed in cm^−1^) is recorded. First, the background spectrum *I*_0_ (cm^−1^) is obtained where all components but the sample of interest are present in the optical path of the spectrometer. In the experiments described in this work, this corresponds to the mounted ATR unit with the active part of the ATR element covered with solvent. Subsequently, the sample, here particles contained in the solvent, is placed on the ATR element, and the spectrum *I* (cm^−1^) is recorded. Calculation of the absorbance spectrum following the Beer–Lambert law

$$A\left(\tilde{\nu }\right)=-\log\frac{I\left(\tilde{\nu }\right)}{I_{0}\left(\tilde{\nu }\right)}$$

yields the specific absorption spectrum of the sample (particles) only, as all other contributions to the achieved light throughput by optical components and solvent cancel out and only the contribution of the particles remains.

An IR absorption spectrum can be acquired in minutes to seconds and delivers specific molecular information about the sample in the optical pathway (Chalmers and Griffiths 2002). In industrial environments it has become state of the art to connect the ATR sensor to the spectrometer with flexible mid-IR conducting fibers. With this technology, the ATR sensor can be immersed in a reactor, thus enabling in-line assessment of the production process.

We set out to exploit the particle manipulation abilities of an USW in ATR spectroscopy. USWs had already been applied with a horizontal ATR unit, attached to a standard FT-IR spectrometer, to improve long-term stability during online bioprocess monitoring (Radel et al. 2010a). In an online configuration, a process stream is continuously taken from the bioreactor and directed to the FT-IR spectrometer. Also, we reported the first successful application of an USW to direct particles of various laboratory suspensions into the evanescent field of an ATR (Lendl et al. 2010).

In continuing these successful attempts to combine an USW and an ATR FT-IR fiberoptic probe, we present our results obtained with prototype setups approaching the research goal of an in-line device, that is, a device in which the ATR sensor is immersed in the bioreactor. With in-line spectroscopy applications in biotechnology in mind, we describe the application on suspensions of *Saccharomyces cerevisiae*, baker's yeast in this work. The choice of yeast as a biological model was guided by availability and simplicity in handling (Hawkes et al. 1997). However, in the context of ultrasonic radiation forces, the spherical shape of the yeast cell is advantageous with respect to the applicability of theoretical results, where spherical particles are usually investigated (Doinikov 1997; Gröschl 1998a; King 1934).

Generally, exposure to an USW does not harm the cells; yeast have been shown to be viable after sonication (Radel et al. 2000b). Moreover, it has been shown that yeast are able to reproduce after ultrasonic arrangement in a cluster (Gherardini et al. 2005). Only when the cells left the pressure nodes of the USW significant alterations in viability were observed (Radel et al. 2000a). Spengler et al. (2000) investigated the effects of primary axial and transverse acoustic radiation forces on yeast in a one-wavelength resonator. They observed a distinct, reproducible pattern of yeast cell clump formation, indicating that the transverse acoustic radiation force (resulting from the energy density distribution over the transducer surface) can have a strong effect on cell distribution. As yeasts are of industrial relevance, reports on the use of ultrasound in respective bioprocesses exist (Chisti 2003; Palme et al. 2010).

**Fig. 1:**
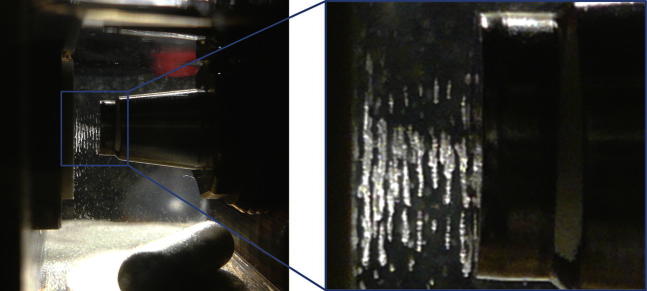
An ultrasonic standing wave is built up between the transducer and the attenuated total reflection (ATR) probe acting as reflector. The enlargement on the right-hand side shows the half-wavelength structure of the particle agglomerates (polystyrene beads were used here for demonstration purposes). By changing the frequency, the location of the pressure nodal planes is altered, and therefore, the evanescent field of the ATR is either populated with particles or depopulated. Taken from: Brandstetter M. Ultrasonically enhanced in-line attenuated total reflection (ATR) infrared absorption spectroscopy of suspensions. Master Thesis, Vienna University of Technology, 2009.

To combine the optical and ultrasonic techniques it was necessary to implement an USW in proximity of the ATR probe tip. One way to accomplish this was to use the ATR probe as the reflector of an ultrasonic resonator. Therefore, the USW was built up between an ultrasonic transducer and an ATR fiber probe. As a consequence, suspended cells agglomerated in the pressure nodal planes, parallel to the surface of the ATR probe (Radel et al. 2010b). As the position of the nodal planes depends on the driving signal, it was expected that the precise location of these cell aggregates relative to the evanescent field could be controlled by the ultrasonic frequency. Figure 1 shows this effect on the example of polystyrene beads in tetrahydrofurane (THF). One can clearly see the particles, which agglomerated in planes. The same behavior was expected for yeast cells in water; however, because of the turbid appearance of yeast suspensions, photographic proof was not feasible.

## Methods

### Experimental setups

Two different setups were investigated: a brass/glass chamber containing the suspension with the ultrasonic field oriented horizontally and an USW equipped in-line probe immersed in a vessel that could be inclined at any angle relative to gravitational forces.

In both cases the ATR probe was connected to the spectrometer by flexible silver halide fibers, and both probes contained an ATR unit at the top. Diamond was used as the ATR element because of its optical properties (refractive index) and inertness. The optical setups provided a bandwidth from 600 to 1960 cm^−1^, a range including the “fingerprint region” most important for the analysis of organic molecules. Spectra were recorded by the accumulation of 32 scans at a spectral resolution of 4 cm^−1^.

For both devices, frequency values for retracting the cells from the ATR (retracting frequency, *f*_*1*_) and pushing them onto the ATR (pushing frequency, *f*_*2*_) were assessed in advance. Successful control over the position of the yeast cells relative to the ATR sensor surface was demonstrated when significant increases and decreases in the measured absorption bands specific to yeast occurred.

#### Chamber setup

**Fig. 2:**
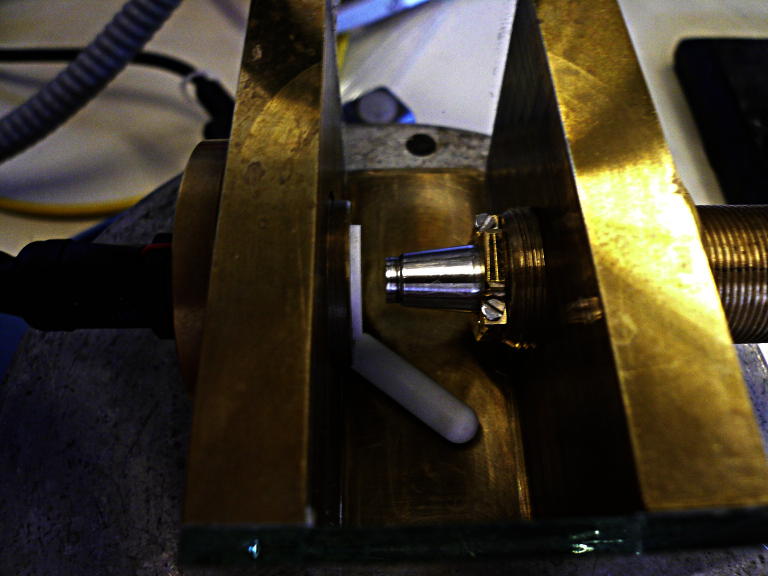
Experimental chamber setup made of brass; on the left hand side the Macor part of the ultrasonic transducer is visible. Facing it, the ATR probe is mounted onto the right wall of the chamber. By turning the fitting, the distance between the probe and the transducer can be adjusted. The white object at the bottom of the chamber is the magnetic stirrer.

The chamber (70 × 40 × 40 mm^3^) consisted of a brass bottom and two brass walls opposite each other. The other two walls were made of glass to allow for visual observation of particle manipulation. The ATR probe was mounted adjustably in an opening in one of the brass walls exactly facing the ultrasonic transducer in the opposite wall (Fig. 2).

A brass fitting with a M20 × 1 fine-pitch outside thread and a 12-mm inner drilling enclosed the ATR probe and maintained proper fixation within the feed-through of the chamber. Hence, by rotation of the brass fitting, the distance between the ATR's front face and the transducer surface could be adjusted; a gap width of 1.6 mm was used for the experiments described here.

The sound source, a piezoelectric sandwich transducer, consisted of a 16-mm-diameter, 1-mm-thick PZT (lead–zirconate–titanate) element equipped with a wrap-around electrode (PZT 181, PI Ceramics, Lederhose, Germany) glued to a carrier. The resonance frequency of this element was approximately 2 MHz. This carrier was made of Macor (Corning, Corning, NY, USA), a glass ceramic that approximately meets the acoustic properties needed for the matching layer and is electrically insulating as necessary. Unlike glass, however, it is machinable and, therefore, more suitable than the glass carriers used before in similar applications (Gröschl 1998b).

The IR spectra in the chamber setup were acquired with a React IR 45 m FT-IR spectrometer and a 9.5-mm DiComp process analysis probe (both from Mettler Toledo, Giessen, Germany).

#### In-line probe

**Fig. 3:**
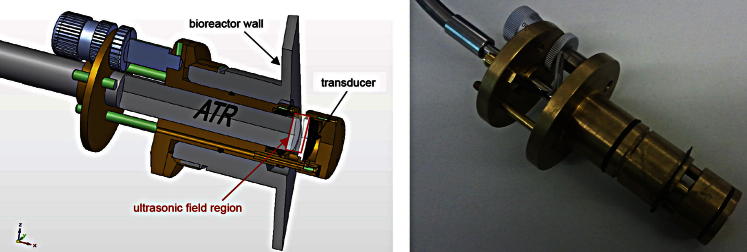
(*Left*) Workshop drawing of the in-line probe connected to the bioreactor. The position of the ATR probe could be adjusted with the micrometer screw gauge along the axis, thus changing the resonator length between ATR probe and transducer. (*Right*) Photograph of the in-line probe.

A prototype in-line probe for use inside a bioreactor was manufactured in house. The probe was designed in accordance with U.S. Food and Drug Administration regulations, and only biocompatible materials (brass, Macor, Viton [DuPont Dow, Wilmington, DE, USA]) were used. The sound source, a 10-mm-diameter, 1-mm-thick PZT transducer (resonance frequency = approximately 2 MHz) equipped with a wraparound electrode (PZT 181, PI Ceramics) glued to a Macor carrier, was held exactly opposite the ATR probe by three rods, one of which acted as a guide for the cable connecting the transducer to the frequency power synthesizer. The distance between the ATR probe and the transducer could be adjusted with a micrometer screw gauge moving the ATR probe along its axis (Fig. 3).

For the experiments described here, the in-line probe was immersed in a beaker equipped with a magnetic stirrer holding the cell suspensions. The angle of the ultrasonic field with respect to gravitational forces could be chosen arbitrarily; here it was set to 135°, with the ATR sensor surface pointing downward.

For the in-line probe, a Matrix M FT-IR spectrometer (Bruker Optics, Ettlingen, Germany) and a 6-mm ATR probe (custom-built, FS, Aachen, Germany) were used.

### Experimental procedure

The equipment (probe, chamber setup, *etc.*) was carefully cleaned with the appropriate solvent (water, ethanol) and dried with paper towels prior to each experiment. A fresh suspension of baker's yeast (dried active yeast, Allinson, Maidenhead, UK) in water was prepared, and the cell concentration was adjusted to approximately 5 × 10^7^ cells/mL for the proof-of-principle experiments. To investigate the concentration dependency of the measurement system, a series of concentrations (14, 17, 20, 24, 29, 35 and 42 g/L dry weight) were measured with the in-line setup. Depending on the setup used, the chamber or the beaker was filled with the cell suspension and stirred at 300–500 rpm with a magnetic stirrer placed on the bottom.

Subsequently, a frequency power synthesizer (FPS2540, Psi, Hinterbrühl, Austria) was switched on. This device was connected to the PZT delivering the driving signal with approximately 1 W true electrical power input; the operating frequency was adjusted in the range 1.8–2 MHz.

As a preliminary proof-of-principle experiment, the appropriate ultrasonic frequencies at which the yeast cells were pushed to and retracted from the ATR respectively were determined. This was done by observation of the FTIR spectra recorded when different ultrasonic frequencies were applied to a yeast suspension. From the change in absorption bands specific for yeast cells, appropriate pushing and retracting frequencies were identified. The location of the nodal planes is influenced by the acoustic wavelength, which, in turn, is a function of the speed of sound and is therefore influenced, for example, by the ambient temperature. Thereafter, when the respective experiments were performed, the ultrasonic field was applied for a series of cycles at the retracting frequency (hereafter *f*_1_) and the pushing frequency (hereafter *f*_2_), respectively. During these sequences, IR spectra were recorded continuously every 10 s for experiments with the chamber setup and every 16 s when the in-line probe was used (the acquisition time is dependent on the optical resolution and differed for the two spectrometers). The change between the frequencies of the ultrasonic field was performed as quickly as possible by turning a knob; typically this was accomplished in less time than it took to acquire one IR spectrum.

Switching off the ultrasonic field concluded the sequence; in the chamber setup a purging step was performed when the IR spectra suggested that some material was attached to the ATR. This was accomplished by driving the transducer at a frequency of about 170 kHz at 1 W true electrical power input.

## Results

### Measurement of reference spectra of yeast

**Fig. 4:**
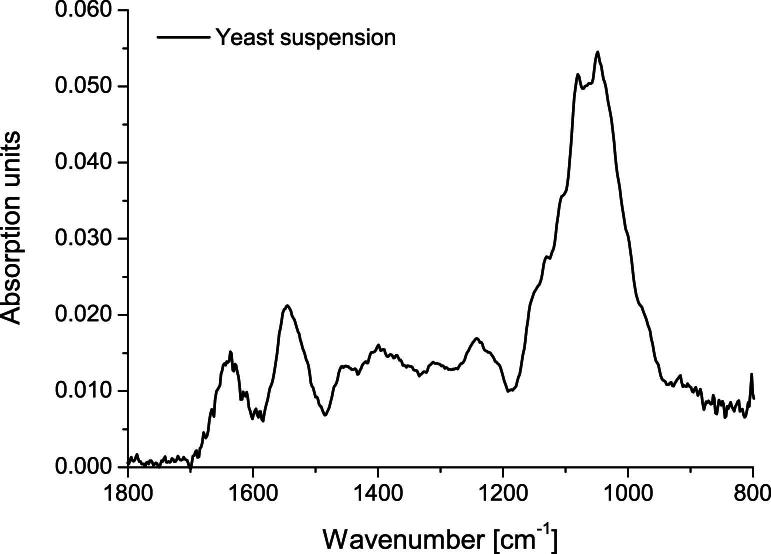
Absorption spectrum of yeast cell sediment on top of the ATR fiber probe tip.

Reference spectra were acquired by letting yeast cells sediment on the tip of the vertically aligned ATR fiber probe. Figure 4 shows the resulting absorption spectra. The bands just below 1650 cm^−1^ and around 1550 cm^−1^ are called amide I and amide II, respectively, and reflect the presence of proteins in the evanescent field of the ATR. The high absorption around 1047 cm^−1^ is caused by the C–O stretching and C–O–H deformation oscillations of the carbohydrates contained in the yeast cells.

### Measurements in chamber setup

Prior to the construction and application of an in-line probe, the brass chamber described before was used to prove that the concept of ultrasound-enhanced ATR spectroscopy of suspensions presented in Radel et al. (2010b) is applicable to yeast cells.

**Fig. 5:**
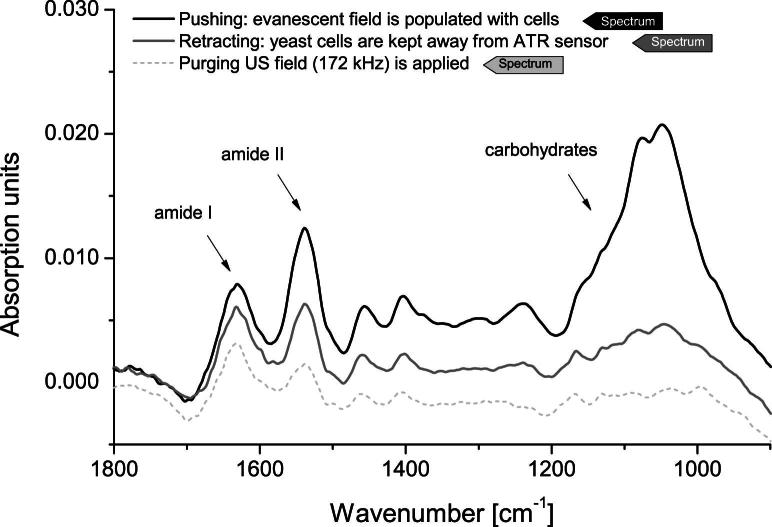
Absorption spectra obtained when the evanescent field was populated with yeast cells (pusing frequency, *black line*) and when it was depopulated (retracting frequency, *gray line*) by the acoustic radiation forces. The dashed line represents the measurement obtained after application of a purge field at 170 kHz at the end of the experiment. The marker arrows in the legend refer to Figure 6.

**Fig. 6:**
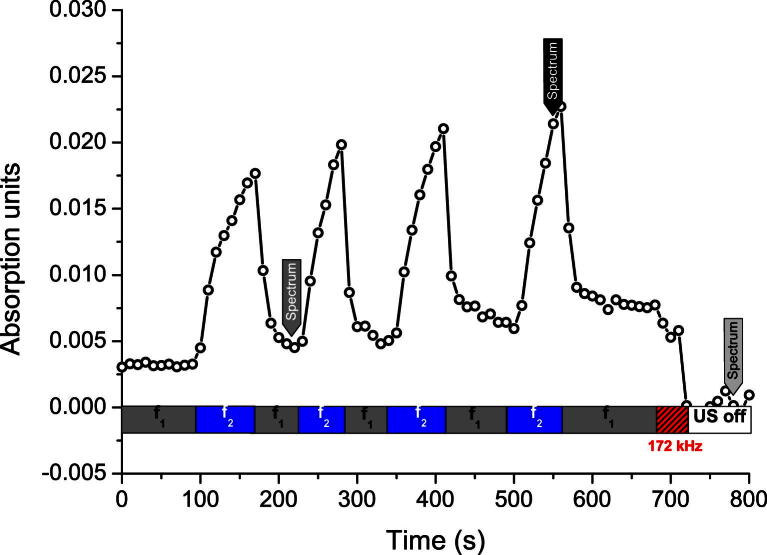
Change in the carbohydrate absorption band at 1047 cm^−1^ during repeated applications of the pushing and retracting frequencies in the chamber setup. The absorption reflects the amount of yeast cells in the evanescent field of the ATR probe.

Figure 5 shows absorption spectra recorded during the experiments in the chamber setup. Spectra of yeast cells could be recorded when the pushing frequency (*f*_2_ = 1.863 MHz) was applied at the time indicated by the black marker arrow in Figure 6.

The gray line in Figure 5 depicts the situation when cells are retracted from the evanescent field of the ATR by the USW at *f*_1_ = 1.878 MHz (indicated by dark gray marker arrow in Fig. 6). A significant decrease in absorption by carbohydrates and amide II was detected. However, the amide I band was not lowered to the same extent. Application of the purge field at 170 kHz somewhat cleaned up the evanescent field region, as suggested by the further decrease in absorption (dashed line).

Figure 6 shows development of the absorption band at 1047 cm^−1^ (carbohydrate) during the sequences of pushing frequency and retracting frequency. Successful population and depopulation of the evanescent field at the surface of the ATR probe are strongly suggested by the steep increase in absorption at the switch to the pushing value *f*_2_. The increase was persistent while the transducer was driven at this frequency; however, the rate of increase decayed. As soon as retracting frequency *f*_1_ was applied, absorption decreased abruptly. Again an ongoing decline was observed; however, it was less pronounced compared with the increase.

Over the course of the experiment, the maximum absorption reached during one cycle increased. Also, the absorption detected in retraction mode at *f*_1_ increased over time, suggesting the occurrence of biofouling on the ATR surface. However, after a short application of the purging frequency (170 kHz) at the end of the experiment, the signal dropped to or even below the initial level, suggesting that the ATR sensor surface was free of cellular material.

Table 1 gives the maximum absorption reached when applying the pushing frequency *f*_2_ and when letting the cells sediment on the ATR element (the same yeast suspension was used). In this setup, absorption using acoustic radiation forces reached about 50% of the maximum absorption when the cells sedimented onto the ATR.

### Measurements with in-line probe

After the successful experiments with the chamber setup, the in-line probe was constructed, assembled and tested. For the in-line probe setup the yeast cells were pushed into the evanescent field of the ATR probe at *f*_2_ = 1.990 MHz and retracted at *f*_1_ = 1.956 MHz. The repeated switching of the USW between pushing and retracting frequency again resulted in distinct increases and decreases in absorption. The absorption measured after the ultrasonic field had been switched off was significantly reduced, indicating that most of the cells had left the evanescent field. The respective maximum absorption values for the same yeast suspension when the cells were allowed to sediment on the ATR element and when measured in-line are given in Table 1. In the presence of ultrasound, the maximum absorption observed was about 30% of the value seen when cells were allowed to sediment on the probe.

**Fig. 7:**
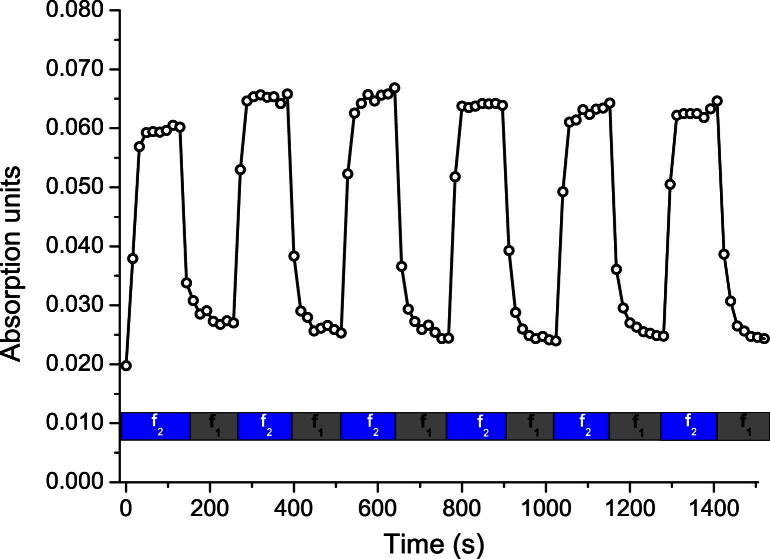
Repeated applications of the pushing (*f*_2_) and the retracting (*f*_1_) frequencies recorded with the in-line probe. Population/depopulation of the ATR is indicated by the temporal development of an absorption band (carbohydrates) of the yeast cells.

The change in the carbohydrate absorption band over time is shown in Figure 7. Pushing frequency *f*_2_ was applied during the recording of eight absorption spectra, where each absorption measurement is represented by a data point in Figure 7; then a switch to retracting frequency *f*_1_ was made and again eight spectra were recorded. This was repeated five times. Absorbance increased rapidly when the switch was made from retracting frequency *f*_1_ (gray bar in Fig. 7) to pushing frequency *f*_2_ (blue bar in Fig. 7). After the switch back to *f*_1_, the signal dropped instantaneously, indicating that the cells were removed from the evanescent field very quickly. The maximum absorption values were seen to increase slightly with successive cycles. When the ultrasonic field was switched off at the end of the experiment, the signal dropped to zero quickly, which was assumed to be caused by gravity; application of the purging frequency was not necessary, in contrast to the chamber setup (Fig. 6).

**Fig. 8:**
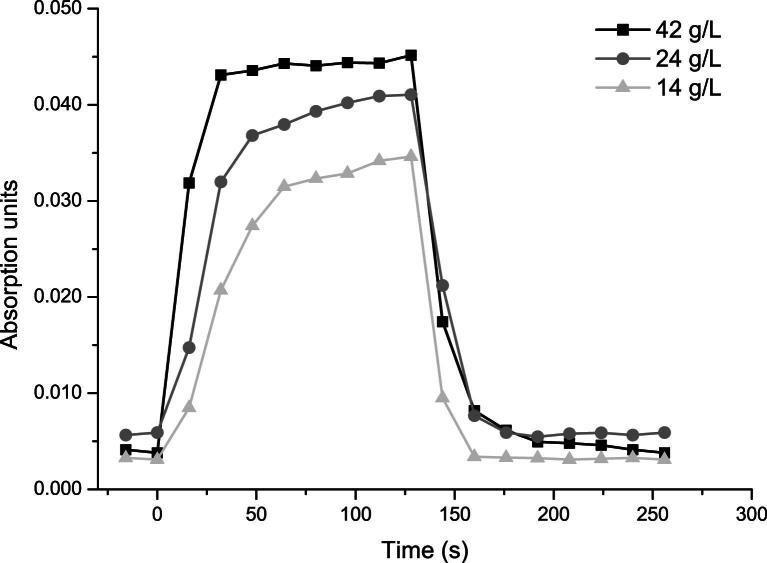
Change in absorption of the carbohydrate band with yeast cell concentration when the pushing frequency is applied (recorded with the in-line probe). Each data point was calculated as the average absorption measured at the time elapsed (Δ*t*) after sonication at pushing frequency; that is, *t* = 0 s corresponds to the last spectrum before application of the pushing frequency, Δ*t* = 16 s is the average of the first spectrum after pushing frequency was switched on, and so on.

Differences in the rate of increase are suggested by the data illustrated in both Figure 6 and Figure 7. An influence of cell concentration was suspected. The measurements shown in Figure 8 were conducted to evaluate the influence of this parameter. Maximum absorption was found to increase with yeast cell concentration, as can be seen in Figure 8. There, the average absorption values recorded for yeast concentrations of 14, 24 and 42 g/L are shown as an example. Moreover, during a pushing cycle, a change in the rate of absorption increase with yeast cell concentration is noticeable. Values close to maximum absorption are reached faster for 42 g/L than for 14 g/L.

**Fig. 9:**
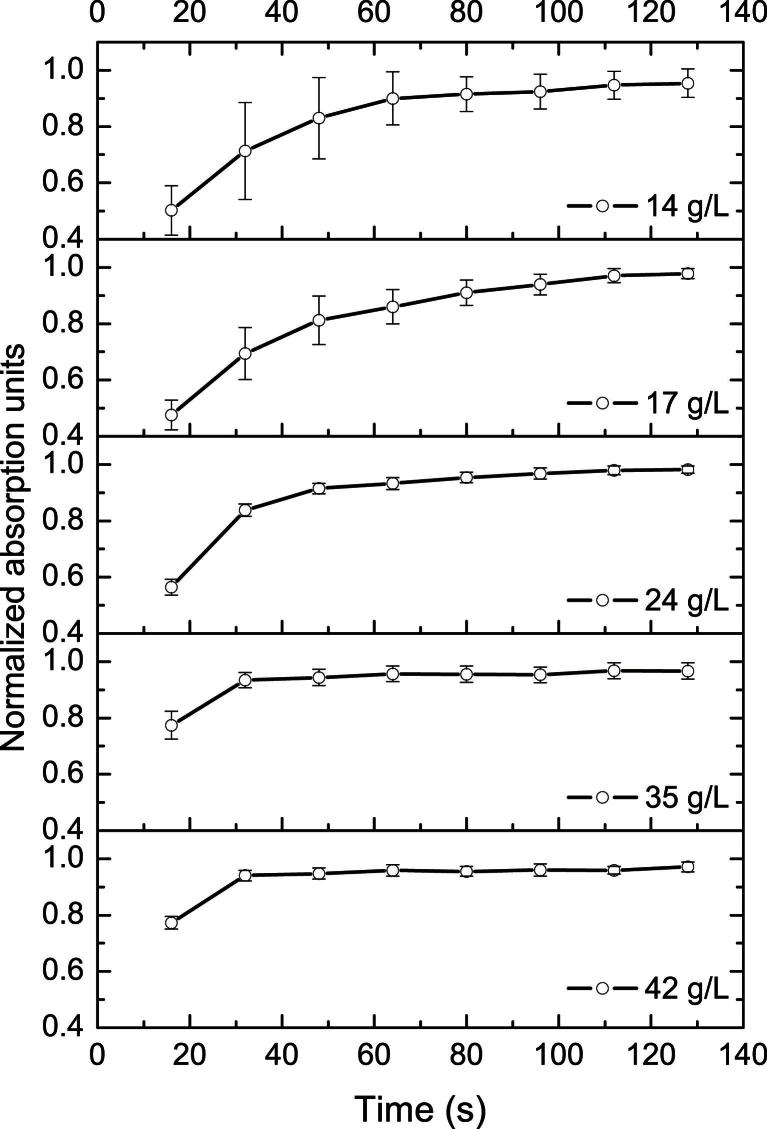
Absorptions normalized to the absolute maximum found for each concentration and averaged over the applications of pushing frequency (averaged over all spectra acquired at Δ*t* = 16 s after application of pushing frequency, at Δ*t* = 32 s, *etc*.) recorded with the in-line probe. Note the change in the rate of increase in relative absorption with changing concentration and the differences in standard deviation with cell concentration stemming from push-to-push variations which are greater for smaller concentrations.

To analyze this change in increase rate further, the absorption values measured at each concentration were normalized to the respective absolute maximum found and the single pushes were averaged. In other words, the respective spectra acquired during application of the pushing frequency *f*_2_ were averaged for each time point of the push: the absorptions acquired immediately after applying *f*_2_ (Δ*t* = 16 s) were averaged, the absorptions found at Δ*t* = 32 s after the switch to *f*_2_ were averaged (*i.e.,* the second spectrum acquired after switching to *f*_2_ for each push), and so on. The first push was omitted because the increase differed from those for the consecutive six pushes for all concentrations. In Figure 9 these data are shown for yeast cell concentrations of 14, 17, 24, 35 and 42 g/L (from top to bottom). The different increase rates are well visible; maximum absorption is reached later at low concentrations (14–24 g/L) than at high concentrations (29–42 g/L). Furthermore, the variations between single pushes are reflected by the standard deviations; low concentrations showed higher push-to-push variations than high concentrations. The standard deviation (*i.e.,* push-to-push variation) for 14 g/L is higher than that for 17 g/L; the same holds true for 35 and 42 g/L.

The average normalized absorption values measured at Δ*t* = 16 s (first spectrum after application of *f*_2_ for pushes 2 to 7) differ significantly from each other for all concentrations (two-sided t-test, α = 0.05), except for the two highest concentrations; the mean absorption found for 35 g/L does not differ significantly from that found for 42 g/L. After Δ*t* = 80 s (fifth spectrum after switching to *f*_2_) no significant difference was found between mean normalized absorptions for all concentrations, indicating that values close to the absolute maximum had been reached by then for all pushes.

## Discussion

For both setups, substantial increases in the observed carbohydrate absorption bands were detected immediately after application of the respective pushing frequency. Cells located in the small volume between the ATR probe and the closest pressure nodal plane were obviously pushed toward the probe tip into the evanescent field region. Subsequently, additional cells were driven into this area because of the agitation of the suspension, leading to an increase in the signal up to a threshold. Moreover, the radiation forces might have increased the cell concentration at the location of sensitivity of the ATR.

With the pushing frequency applied, the rate of increase in absorption was observed for both experimental setups. In the chamber setup, the slopes appeared to be steeper. This could indicate an influence of the angle between the gravitational forces and the axial ultrasonic radiation force. This angle was different for the two setups, with a higher net pushing force for the chamber setup (90° between ultrasonic radiation force and gravitational force) than for the in-line probe (135° between the respective forces).

The absorption achieved when cells were actively pushed into the evanescent field by the USW was compared with the absorption reached by simple sedimentation of yeast cells on the ATR. It revealed that approximately 50% of the achievable absorption of the sediment was reached in the chamber setup and roughly 30% in the in-line probe, respectively. The reason for this difference might again be that the ultrasonic field was horizontal in the chamber setup, whereas the radiation forces had to push the cells 135° upward against gravity in the case of the in-line probe.

The increase in the peak value of absorption over several cycles of switching between retracting and pushing frequency is larger for the in-line probe than for the chamber setup. This could be attributed in part to the duration of the application of the retracting frequency at the beginning of the experiment. This period lasted about 180 s in the chamber setup, but only about 130 s for the in-line probe. During this time cells were possibly “pre”-agglomerating in the pressure nodal planes outside the evanescent field.

Measurements of different yeast cell concentrations with the in-line probe showed that high concentrations led to very fast increases in absorption toward the maximum value observed; for low cell concentrations, this increase was found to be slower and more variable throughout consecutive applications of pushing frequency *f*_2_. This is most likely dependent on the number of cells available to contribute to mid-IR absorption, which is of course concentration dependent.

## Conclusions

In this first step toward an inline ATR FT-IR spectroscopy probe for fermentation monitoring, we could successfully record spectra of yeast cells in stirred suspensions by applying ultrasonic radiation forces for particle manipulation. Yeast cells could in this way actively be forced into and out of the evanescent field region of an ATR probe. Hence, this ultrasound-enhanced fiberoptic probe enabled measurement of cells and supernatant separately. Furthermore, it was observed that the rate of increase in yeast-specific absorption bands during application of an USW at the pushing frequency depended on the cell concentration in the suspension. At all concentrations investigated, 90% of the maximum absorption measured was reached within 32 s. This would allow measurement intervals that are sufficiently short for quasi-real-time in-line fermentation monitoring.

## Figures and Tables

**Table 1:** Maximum absorption found when applying the pushing frequency and when letting the cells sediment onto the ATR element of the respective setup.

Setup	Maximum absorption (mAU)	
	Ultrasound	Sedimentation
Chamber	23	54
In-line probe	46	164
